# In Silico Drug Repurposing for SARS-CoV-2 Main Proteinase and Spike Proteins

**DOI:** 10.1021/acs.jproteome.0c00383

**Published:** 2020-09-07

**Authors:** Irene Maffucci, Alessandro Contini

**Affiliations:** † Université de technologie de Compiègne, UPJV, CNRS, Enzyme and Cell Engineering, Centre de recherche Royallieu - CS 60 319 - 60 203 Compiègne Cedex, France; ‡ Università degli Studi di Milano, Dipartimento di Scienze Farmaceutiche, Sezione di Chimica Generale e Organica “A. Marchesini”, Via Venezian, 21 20133 Milano, Italy

**Keywords:** SARS-CoV-2, COVID-19, virtual screening, main proteinase, 3CLpro, spike protein, molecular dynamics, MM-GBSA, drug repurposing

## Abstract

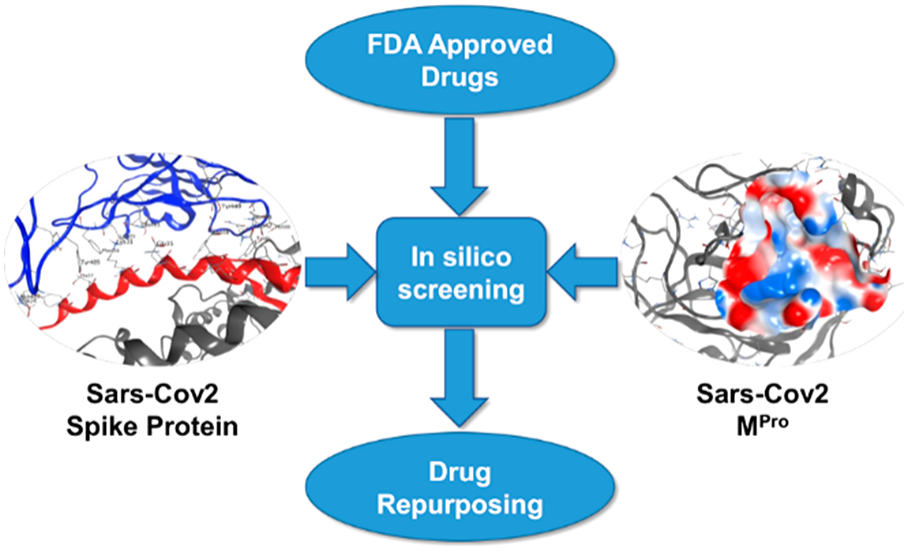

The pandemic caused by SARS-CoV-2 is currently representing a major health and economic threat to humanity. So far, no specific treatment to this viral infection has been developed and the emergency still requires an efficient intervention. In this work, we used virtual screening to facilitate drug repurposing against SARS-CoV-2, targeting viral main proteinase and spike protein with 3000 existing drugs. We used a protocol based on a docking step followed by a short molecular dynamic simulation and rescoring by the Nwat-MMGBSA approach. Our results provide suggestions for prioritizing *in vitro* and/or *in vivo* tests of already available compounds.

## Introduction

The outbreak of a novel β-coronavirus, SARS-CoV-2, is currently a pandemic threat, with already more than 23 million confirmed cases and more than 800 000 deaths all over the world, according to the World Health Organization (data of August 2020, https://covid19.who.int). Unfortunately, although many clinical and preclinical studies are ongoing, to date there is not a validated treatment to this infection.

As for other known coronaviruses, such as SARS-CoV and MERS-CoV, the SARS-CoV-2 entry into host cells is mediated by its transmembrane spike glycoprotein (S-protein). This is a trimeric protein belonging to the class I fusion proteins, whose structure for SARS-CoV-2 has been partially resolved by cryo-electron microscopy (code PDB 6VXX and 6VSB).^1,2^ The S-protein is divided into two functional subunits: the S_1_ subunit, which contains the receptor binding domain (RBD) responsible for the interaction with host cell’s receptors, and the S_2_ subunit, which is implicated in the fusion of the viral and cellular membranes.

Recent works showed that SARS-CoV-2 S-protein is able to bind the human angiotensin-converting enzyme 2 (hACE2),^3−5^ explaining the symptoms linked to the SARS-CoV-2 infection (COVID-19), since hACE2 is widely expressed in endothelial cells from small and large arteries, in lung alveolar epithelial cells, but also in the heart, kidney, testis, and gastrointestinal system.^6,7^ Moreover, the crystallographic structure of the RBD in complex with the hACE2 has been recently resolved (PDB code 6M0J),^4^ giving molecular details about this interaction (Figure 2). The binding to the host cell receptor triggers a series of conformational changes which allow the fusion with the host cell and the entry of the virus.^1^

In addition, a recognized target for coronaviruses treatments is the main proteinase M^pro^, also known as 3CL^pro^.^8,9^ This protein processes the polyprotein 1ab into mature nonstructural proteins that are essential for viral replication^10^ and is rather conserved among coronaviruses. Moreover, human proteases with the same specificity have not been discovered so far, making M^pro^ an ideal target to treat coronavirus infections. The crystal structure of the SARS-CoV-2 M^pro^ in complex with a covalent peptidomimetic inhibitor (PDB code 6LU7(11)) was made available. Additionally, the Zhang group, developer of the popular homology-modeling software I-TASSER,^12^ made available 24 3D structural models^13^ of proteins in the SARS-CoV-2 genome.^14^ Among these, the model of the M^pro^ (code QHD43415) was made available before the release of the crystal and was characterized by a very high reliability score (TM-score = 0.96).

It is clear that both spike and M^pro^ proteins represent potential targets for anti-SARS-CoV-2 drugs: on one side, hampering the interaction between hACE2 and the viral RBD will block the entry of the virus into the human cells. On the other side, inhibiting the viral proteases, as done with many antiviral drugs currently used in the therapy of HIV infection,^15^ will interfere with the viral replication.

However, the experimental procedure to conceive a new drug is long (up to decades) and expensive (up to several millions of dollars). Such a time and resources price is not affordable in the current emergency situation; therefore, a promising alternative consists in a drug repurposing investigation exploiting *in silico* techniques, such as Virtual Screening (VS), which already proved to be able to identify active molecules against a target.^16,17^

Within this context and aiming to give our contribution to the current sanitary crisis, we designed a VS campaign of currently worldwide approved drugs. Despite the fact that similar studies have been recently published,^18,19^ in this work we independently screened more than 3000 molecules against the two SARS-CoV-2 proteins mentioned above to provide information useful for a multiple treatment approach. In addition, we applied a solid VS procedure we recently developed and which was shown to be successful in discriminating active from inactive compounds within the screening of classical small molecules and protein–protein interaction inhibitors.^20^

## Methods

### Receptor Preparation

Receptor models for the SARS-CoV-2 M^pro^ were prepared starting from both the 6LU7 crystal structure and the QHD43415 I-Tasser model. This choice was made to take binding site flexibility into account through an ensemble docking approach^21^ but without the need to perform time-consuming molecular dynamic (MD) simulations to generate reliable conformational ensembles. The two M^pro^ models were prepared using the MOE2019 software,^22^ with the following protocol:

6LU7: all water molecules were deleted. The covalently bound peptidomimetic ligand was then unbound from Cys145, and the α,β double bond of the ligand, that behaves as a Michael acceptor, was restored. The Structure Preparation module of MOE was used to correct PDB inconsistencies and to assign the protonation state at pH = 7.0. The default Amber10EHT force field, coupled to the Born solvation model was assigned to the system. The ligand was then minimized, keeping the receptor constrained. Then, the receptor was minimized by applying backbone restraints and keeping the ligand constrained. Finally, the complex was minimized in two separate steps, first by keeping backbone restraints, second by removing all restraints. All minimizations were performed up to a gradient of 0.1 kcal mol^–1^ Å^–2^. The receptor and the ligand were then saved for future use.

4MDS: the crystal structure of SARS-CoV 3CL^pro^ proteinase,^23^ a close homologue of SARS-CoV-2 M^pro^, in complex with a carboxamide inhibitor was also modeled to be used as an additional reference; this was done because no specific SARS-Cov2M^pro^ noncovalent inhibitors were published at the time of this screening.^24^ The system was prepared for calculations as follow: the PDB was corrected and protonated at pH = 7.0 using MOE as stated above. The ligand was minimized, keeping the receptor constrained, using the MMFF94x force field coupled with the Born solvation model. The receptor was then minimized, keeping the ligand constrained, using Amber10EHT+Born. Finally, the complex was minimized in two steps, as described above.

QHD43415: the I-TASSER model QHD43415_5^13^ was superposed to 4MDS (prepared as previously described) in order to precisely define the binding site. Since we observed that the 4MDS ligand also fitted QHD43415_5, the ligand was transferred, and the complex was prepared as described for 4MDS. The resulting structure was used for docking.

The RBD of the S-protein was obtained by the recently resolved X-ray structure of the complex between the SARS-CoV-2 RBD and the human ACE2 (code PDB 6M0J).^4^ After the deletion of this latter, the RBD has been protonated at physiological conditions using the H++ server.^25^

### RBD binding site definition

In order to determine the RBD residues playing the most important role in the binding to ACE2 (hot spots), the complex between RBD and ACE2 has been initially protonated as the single RBD. Successively, it has been submitted to a molecular dynamics (MD) simulation using the AMBER18^26^ package and the ff14SB^27^ force field. The system has been neutralized by adding the proper number of Na^+^ ions and solvated adding a cubic box of TIP3P water up to a distance of 10 Å from the solute. The system has been relaxed by optimizing the geometry of hydrogens, ions, and water molecules (1000 cycles of steepest descent and 4000 cycles of conjugated gradient). The solvent box has been equilibrated at 300 K by 100 ps of NVT (constant volume and temperature) and 100 ps of NPT (constant pressure and temperature) simulation. Then, a minimization of side chains, water, and ions (2500 cycles of steepest descent and 2500 cycles of conjugated gradient) and a global minimization (2500 cycles of steepest descent and 2500 cycles of conjugated gradient) were performed with a restraint of 10 kcal/mol applied on the backbone atoms. Successively the system has been heated up to 300 K in 6 steps of 20 ps each (Δ*T* = 50 K) during which the backbone restraints were reduced progressively from 10 to 5 kcal/mol. The systems were then equilibrated for 100 ps in the NVT ensemble and for 200 ps in the NPT ensemble keeping a 5 kcal/mol restraint on the backbone atoms. This was followed by a 4 steps NPT equilibration during which the restraints were progressively reduced to 1 kcal/mol. Finally, after a 500 ps unrestrained NPT equilibration, a production run of 20 ns was performed. During the whole simulation, an electrostatic cutoff of 8 Å, a time step of 2 fs, and the SHAKE algorithm were applied.^28^ The root mean squared deviation (RMSD) of the backbone atoms using the X-ray structure as reference was used as a metric of simulation convergence (Figure S1). Hydrogen bonds (H-bond) analysis was performed on the last 10 ns of the simulation using the cpptraj module of AmberTools and using a donor–acceptor distance cutoff of 3.5 Å and a donor–donor hydrogen-acceptor angle cutoff of 150 deg (Table S2).

After having defined as interfacial those RBD residues whose difference in the solvent accessible area when going from the complex to the isolated state was greater than 0.75 Å, an *in silico* alanine scanning was performed on the last 10 ns of the production run. The mutated complexes have been built using PyMol,^29^ and the alanine scanning was run with the Amber mmpbsa.py code on one frame every 100 ps and by choosing the GB-Neck2^30^ as implicit solvent model (igb = 8), the mbondi2 as radii set, and a salt concentration of 0.15 M. The ΔΔ*G* was calculated as the difference between the Δ*G* of the mutated system and the one of the native system (Table S1). The residues giving ΔΔ*G* greater than 2.5 kcal/mol were considered as hot spots and were used to define the RBD potential binding site for small molecules.

### Database Preparation

Two separate databases were downloaded from the corresponding sources^31,32^ and merged. The database was then checked and redundant molecules, identified by CAS number, were removed. The database was then processed by MOE in order to build the 3D structures and to minimize the geometry of each molecule. The *wash* function of the MOE database tool was used with the MMFF94x+Born force field, requesting the dominant protonation state at pH = 7.0 and preserving existing chirality. The final database, consisting of 3118 unique molecules, was saved in SDF format.

### Virtual Screening

The Virtual Screening (VS) was done according to our recently developed protocol.^20^ This is applied using a set of scripts (available for download as Supporting Information within ref (20)) that does the following steps automatically: (1) Preparation of the screening library, including the generation of tautomers, alternative protonation states, stereoisomers and ring conformers, if requested. (2) Docking of all molecules using PLANTS.^33^ (3) Analysis of results. (4) Parameterization of docked ligands selected for rescoring. (5) Molecular dynamics of complexes selected for rescoring, using Amber.^26^ Rescoring using the Nwat-MMGBSA method.^20,34,35^ All dockings were performed by PLANTS, requesting a search speed = 1 (maximum accuracy) and the ChemPLP scoring function.^36^ Only the principal tautomer and protonation state predicted at pH = 7 were considered for the docking. The following receptor-specific parameters were also set up: 6LU7: binding site center (b.s.c.; x,y,z) = −10.2858, 12.3088, 69.3271; binding site radius (b.s.r.; Å) = 16. QHD43415: b.s.c. = −15.124, 15.0521, −24.6152; b.s.r. = 14. RBD-BS1: b.s.c. = −38.621, 39.731, 1.564; b.s.r. = 17. RBD-BS2: b.s.c. = −36.355, 20.471, 2.322; b.s.r. = 17.

Nwat-MMGBSA rescoring was requested for the top 2% of compounds (about 60 molecules for each target). Rescoring consists in performing a short MD simulation (about 2.5 ns, including 1.5 ns of equilibration and 1 ns of production), followed by calculation of binding energy by MMGBSA.^37^ In a previous work we demonstrated that longer MD simulations are not necessary for this purpose.^20^ Nwat-MMGBSA binding energies were computed by including no explicit waters (Nwat = 0, corresponding to standard MMGBSA calculations) or by selecting a certain number of explicit waters to be included in the calculation (Nwat = 10, 20, 30, 60, and 100).

The same protocol was applied to 4MDS also, since the binding energy computed for the 4MDS crystallographic ligand was used as a reference. Analogously, the binding energy of the 6LU7 ligand (whose covalent bond was broken as described above), was computed as a reference.

## Results and Discussion

### Virtual Screening on M^pro^

The VS campaign on SARS-CoV-2 M^pro^ was conducted on two different models (e.g., 6LU7 and QHD43415) to take binding site flexibility into account through an ensemble docking approach, increasing the solidity of the procedure with respect to previous VSs on the same protein.

The results of the VS campaign are summarized in Table 1 and Table 2, respectively, while Tables S2 and S3, Supporting Information, report compounds selected by docking but that failed during the MD/Nwat-MMGBSA rescoring step. Results of Nwat-MMGBSA rescoring, using 30 explicit waters, are the only reported, since Nwat = 30 was considered a reasonable value in previous publications.^20,34^

**Table 1:** Results of the VS Campaign on the Crystal Structure of SARS-CoV-2 M^pro^ (6LU7)^a^

Drug Name	Dock score	Nwat-MMGBSA^b^
**Angiotensin II**	–124.4	–120.3 ± 10.1
**GHRP-2**	–132.6	–106.0 ± 8.3
**Indinavir**	–122.4	–86.5 ± 5.7
**Polymyxin B**	–107.9	–84.2 ± 8.3
**Fexofenadine**	–107.8	–77.0 ± 7.8
**Atazanavir**	–109.6	–73.0 ± 7.6
**Cobicistat**	–124.3	–72.8 ± 8.3
**Aliskiren**	–109.9	–70.9 ± 6.5
Lercanidipine	–106.6	–67.4 ± 8.4
Darunavir	–108.1	–66.6 ± 6.8
Montelukast	–112.8	–54.9 ± 6.8
Latanoprost	–108.5	–52.5 ± 4.2
Octenidine	–114.0	–50.8 ± 4.9
Velpatasvir	–108.4	–46.5 ± 8.1
Tyloxapol	–112.3	–42.5 ± 6.5
Salvianolic acid B	–124.4	–41.1 ± 11.0
Nilotinib	–106.6	–40.1 ± 8.6
Siponimod	–105.9	–38.5 ± 6.0
Travoprost	–114.9	–35.6 ± 6.1
Vitamin A Palmitate	–107.6	–35.5 ± 6.1
Penfluridol	–110.1	–30.2 ± 7.3
Clindamycin	–106.2	–20.5 ± 15.4
Ledipasvir	–109.6	–20.1 ± 7.8
Elbasvir	–106.3	–19.8 ± 9.9

a Top 2% of compounds selected from the docking of 3118 FDA approved drugs and rescored by Nwat-MMGBSA (Nwat = 30) are shown. Compounds that ranked better than the reference are highlighted in bold. The 6LU7 crystallographic ligand of the SARS-CoV-2 main protease (6LU7) was used as the reference. Docking and Nwat-MMGBSA scores are −132.7 and −70.6 ± 8.0 kcal/mol, respectively.

b Nwat-MMGBSA rescoring was done considering 30 explicit water molecules around the ligand (Nwat = 30).

**Table 2:** Results of the VS Campaign on the Homology Model of SARS-CoV-2 M^pro^ (QHD43415)^a^^,^^b^

Drug Name	Dock score	Nwat-MMGBSA^c^^,d^
**Caspofungin**	–108.3	–97.9 ± 12.4
**Lopinavir**	–106.5	–89.9 ± 5.9
**Atazanavir**	–109.9	–86.0 ± 7.0
**GHRP-2**	–116.7	–79.2 ± 11.1
**Indinavir**	–105.4	–78.6 ± 6.5
**Angiotensin II**	–125.7	–75.7 ± 9.2
**Dehydroandrographolide Succinate**	–99.4	**–61.1**
Ritonavir	–112.3	–58.3 ± 7.8
Azilsartan medoxomil	–102.1	–54.4
Salvianolic acid B	–116.0	–51.0 ± 7.7
Vilanterol	–100.7	–50.9
Elbasvir	–110.2	–48.0 ± 7.7
Clindamycin	–99.6	–47.8
Montelukast	–110.1	–47.5 ± 6.9
Latanoprost	–101.0	–46.8
Cobicistat	–119.3	–45.4 ± 11.6
Octenidine	–104.8	–43.6
Mupirocin	–98.1	–42.3
Tyloxapol	–105.5	–41.1 ± 8.3
Echinacoside	–103.1	–40.0
Salmeterol Xinafoate	–105.3	–37.9 ± 7.3
Ledipasvir	–101.5	–37.3
Thonzonium Bromide	–99.3	–36.7
Lomitapide	–98.1	–34.2
Travoprost	–99.2	–34.0
Itraconazole	–100.2	–32.6
Penfluridol	–106.2	–31.8 ± 9.6
Cisatracurium besylate	–100.3	–23.6
Retinol palmitate	–100.1	–21.8
Terfenadine	–98.1	–17.7

a The homology model of SARS-CoV-2 M^pro^ was made available by the Zhang group at https://zhanglab.ccmb.med.umich.edu/C-I-TASSER/2019-nCov/

b Top 2% of compounds selected from the docking of 3118 FDA approved drugs and rescored by Nwat-MMGBSA (Nwat = 30) are shown. Compounds that ranked better than the reference are highlighted in bold. The 4MDS crystallographic ligand in complex with SARS-CoV 3CLpro, a close homologue of SARS-Cov-2 M^pro^, was used to compute reference scorings. Docking and Nwat-MMGBSA scores are −96.4 and −59.8 ± 5.3 kcal/mol, respectively.

c Nwat-MMGBSA rescoring was done considering 30 explicit water molecules around the ligand (Nwat = 30).

**Figure 1:**
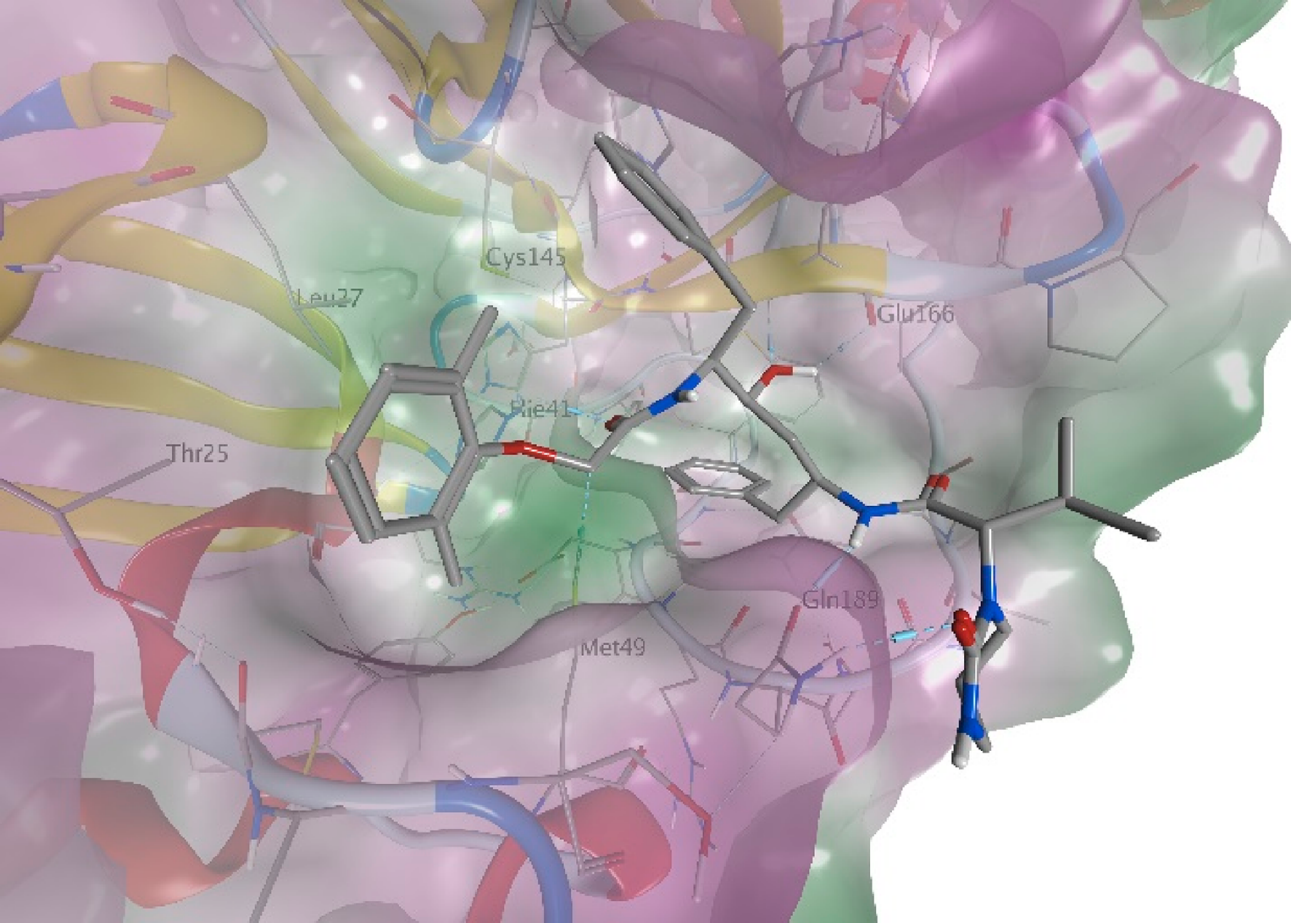
Predicted binding mode of lopinavir to M^pro^. The model was obtained by performing a cluster analysis of the MD trajectory of the docking pose, followed by a backbone-restrained geometry minimization of the main cluster using MOE.

As can be observed, the protease inhibitors indinavir and atazanavir, currently used to treat HIV infections, have been selected by both models. Conversely, the protease inhibitor lopinavir is top ranked for the QHD43415 model only, while it failed during MD for 6LU7 (Table S2, SI), probably due to steric clashes originating from a position of the side chains in the crystal structure not favorable for a stable binding of this compound. Figure 1 shows the predicted binding mode for lopinavir, that anchors to the M^pro^ binding site by multiple H-bonds. The first H-bond is observed between the catalytic His41 residue and the aryloxyacetylamido carbonyl (H-bond length = 2.1 Å). Additional H-bonds are observed with Glu166 and the hydroxyl group at C-4, that acts both as a donor and an acceptor (lop-O(H)···(H)N-Glu166 and lop-OH···O=C-Glu166 distances = 1.8 Å and 2.2, respectively). Finally, a dual H-bond is observed between the side chain of Gln189 and both the butanamido NH (lop-NH···O=C-Gln189 = 1.9 Å) and the 2-oxo-1,3-diazinanyl carbonyl (lop-C=O···H_2_N-Gln189 = 2.0 Å).

In addition, within an *in vitro* study against SARS-CoV-2, it has been shown that lopinavir has an estimated 50% effective concentration (EC_50_) of 26.63 μM in Vero E6 cells.^38^

Other HIV protease inhibitors such as darunavir and ritonavir were selected by one of the models, but failed for the other. Indeed, darunavir scored rather well within 6LU7 screening, while ritonavir was high-ranked by QHD43415 although the Nwat-MMGBSA score was slightly lower than the chosen thresholds for both compounds. Interestingly, similar results were also obtained by another group using artificial intelligence.^39^ However, ritonavir alone showed an EC_50_ greater than 100 μM in Vero E6 cells.^38^

Although some clinical studies on the use of HIV protease inhibitors in COVID-19 were already terminated when this screening was made, their results were not already available (https://clinicaltrials.gov/ct2/results?cond=COVID-19). Now, a publication showing that a combination of lopinavir and ritonavir succeeded in alleviating symptoms and shortening the hospitalization in patients with mild to moderate COVID-19, especially when used in association with ribavirin.^40^ Nevertheless, it should also be noted that no benefits were observed for hospitalized patients with severe COVID-19 when treated with lopinavir–ritonavir, beyond standard care,^41^ suggesting that the treatment is only effective when given at an early stage of the disease. Cobicistat, another drug that is approved for the treatment of HIV infection, has been selected by the VS on the 6LU7 receptor model as a potential M^pro^ inhibitor, even if its main mechanism is claimed to be the inhibition of CYP3A.^42^ At the moment, a combination of darunavir and cobicistat is under clinical evaluation for COVID-19, but preliminary results on efficacy and safety are not encouraging. However, final results are expected for August 31st, 2020.^43^

Some drugs already approved for the treatment of hepatitis C were also identified. These includes elbasvir,^44^ ledipasvir,^45^ and velpatasvir,^46^ that were also identified in other *in silico* screenings.^19,47^ Notably, clinical trials to evaluate the efficacy of ledipasvir on COVID-19 are currently ongoing (https://clinicaltrials.gov/ct2/results?cond=COVID-19).^48^

Interestingly, angiotensin II and GHRP-2 are selected in both screenings. Since the M^pro^ catalytic activity is to cleave the polyprotein, a peptide of about 15 amino acids, it is plausible that peptides, such as angiotensin II and GHRP-2 of 8 and 6 amino acids, respectively, can fit into the M^pro^ active site. Although the use of these hormones might not be indicated for the treatment of SARS-CoV-2, their structures might be used as templates for further drug development. Similarly, the antibiotic polymyxin B was also picked as a high-rank hit, although by the 6LU7 model only, probably due to its peptide nature. Interestingly, polymyxin B was top-ranked within the S-protein screening also, as discussed later in this article. Another lipopeptide, caspofungin, has also been identified, but by the QHD43415 model only. Caspofungin is an antifungal drug specifically used in HIV-infected individuals.^49^ It was also identified in another independent study as an inhibitor of SARS-CoV-2 replication,^50^ even if the Nsp12 polymerase has been claimed as the target.

A hit that might be worthy of attention, although only identified by the QHD43415 model, is dehydroandrographolide succinate (DAS). DAS is a natural product extracted from *Andrographis paniculate*, well-known by traditional Chinese medicine.^51^ Indeed, while the herb has long been used to treat cold and fever, purified andrographolides, including DAS and analogues, have been prepared and used to treat respiratory diseases.^52,53^ Antibacterial, antiviral, anti-inflammatory, and immune-stimulatory activities were claimed for DAS,^54^ including the inhibition of HIV and H5N1 viruses *in vitro*.^55,56^

Several other compounds were selected by initial docking but failed during the MD simulation phase (Tables S2 and S3). Such a failure might be due to several causes, among all poor parametrization (the BCC charge parametrization method,^57^ instead of the more rigorous RESP method,^58^ was chosen for time constraints). However, the reason for the failure might also be due to severe steric clashes or mispositioning during the docking stage. Thus, although some good hits might be found within the “F” series, these selections are to be considered as the least reliable.

### Virtual Screening on Spike Protein

When this work was realized, no S-protein RBD-targeting small molecule was known either for SARS-CoV-2 or other coronaviruses. Conversely, few antibodies recognizing the RBD^59−62^ and a recombinant ACE2 enzyme^63^ are reported. In addition, a 23-mer peptide derived from the ACE2 showed an affinity of 47 nM toward the SARS-CoV-2 RBD,^64^ and a few other peptides were developed to inhibit the interactions of the S2 subunit during the fusion process in both SARS-CoV and SARS-CoV-2.^65^ Nonetheless, considering that several compounds that are currently being tested, some with positive results, were identified by our VS campaign on M^pro^, the results reported hereafter can also represent a step torward the treatment against SARS-CoV-2.

### RBD Binding Sites Definition

**Figure 2:**
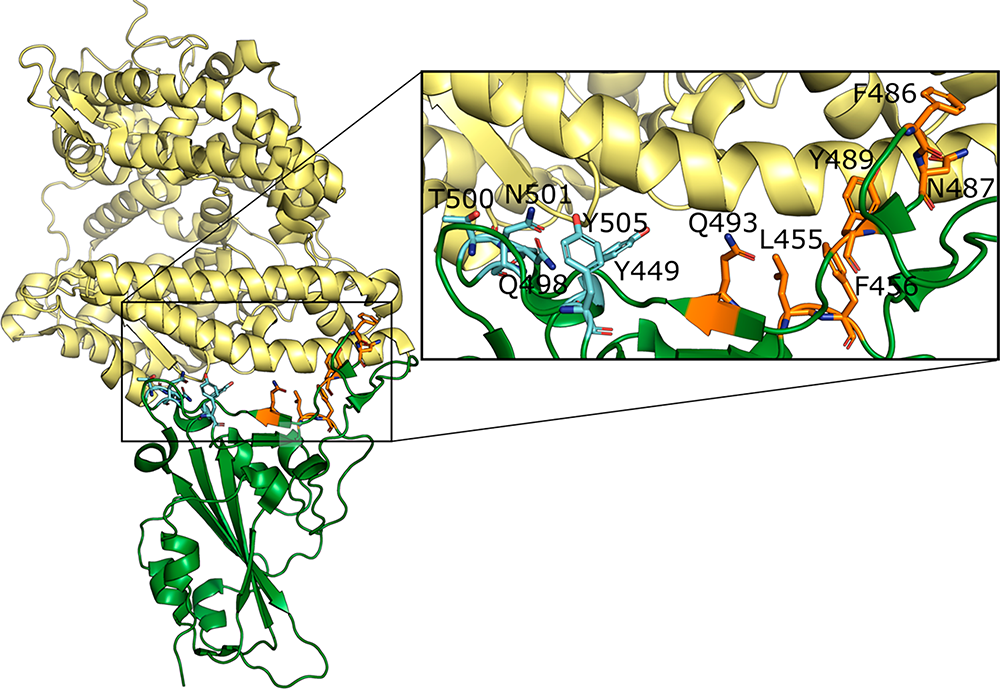
Complex between hACE2 (yellow) and SARS-CoV-2 RBD (green) from the X-ray structure (PDB code 6M0J). The hot spot residues are represented in sticks and labeled in the inset. Hot spots of cluster 1 are represented in orange, while those of cluster 2 are represented in cyan.

**Figure 3:**
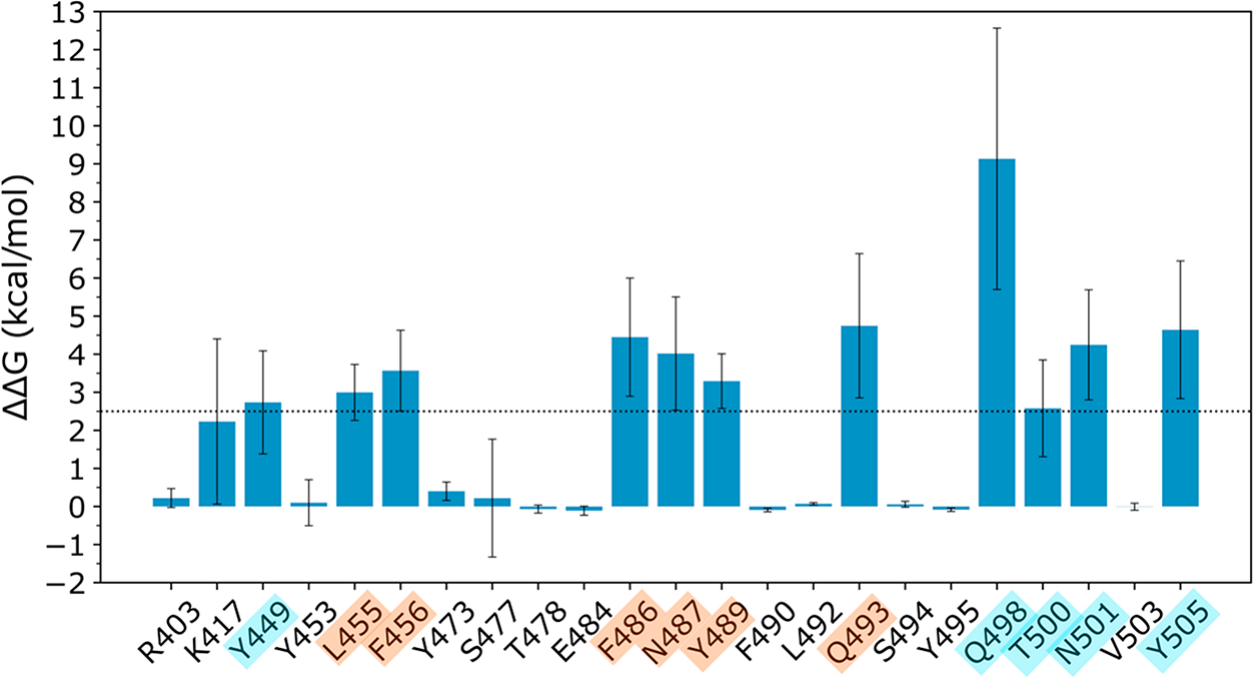
Difference in the binding free energy between the mutated system and the native one computed on the last 10 ns of the MD simulation of the hACE2-RBD complex. All the residues for which the ΔΔ*G* is greater than the threshold (2.5 kcal/mol) have been considered as hot spots. The hot spots of cluster 1 are highlighted in orange and those of cluster 2 in cyan.

As for most of the protein–protein interaction (PPIs) interfaces,^66^ the one between hACE2 and RBD is quite extended. However, it is possible to target only the residues making the major contribution to the binding free energy between the two proteins (hot spots). In light of this, an alanine scanning analysis was performed by individually mutating the RBD residues at the interface with hACE2 (see Methods, Figure 2 and Table S4), in order to determine which are the RBD hot spot residues. This allowed us to define two clusters of hot spots (Figures 2 and 3), in agreement with a recently published preprint article.^67^ More in detail, the first cluster (cluster 1) involves Leu455, Phe456, Phe486, Asn487, Tyr489, and Gln493, while the second one (cluster 2) includes Tyr449, Gln498, Thr500, Asn501, and Tyr505 (Figures 2 and 3). It has to be noted that the found hot spots are not included in the observed mutations found so far,^68^ suggesting that they can be safely targeted by potential inhibitors of the ACE2-RBD interaction.

In the second cluster we can find Gln498: when it is mutated to alanine, the complex Δ*G* has a loss of ∼9 kcal/mol, indicating that this residue is fundamental for the interaction with hACE2. Indeed, the Gln498 side chain is involved in a highly stable H-bond (occupancy >80%, Table S2) with the hACE2 Asp38 side chain, and for the remaining ∼20% it interacts with the hACE2 Lys353 side chain.

Since cluster 1 and 2 are well separated and localized at the two extremities of the RBD interface to hACE2, it is possible to determine two distinct binding sites to target by VS (see Methods), called BS1 and BS2, respectively, in the following discussion. Working with two different possible RBD binding sites represents an advantage as compared to other similar studies, where a single but larger binding site on the RBD was defined.^18,69^ Indeed, as previously said, most of the currently available drugs are small molecules which are able to interact with a limited surface and only with a few residues. Therefore, selecting two binding sites corresponding to specific hot spot clusters makes the docking pose search more efficient, by limiting the search space. In addition, it allows us to discard molecules which are predicted to strongly interact with the protein target but on a protein region without hot spots, not assuring the PPI inhibition.

### Virtual Screening on RBD-BS1

The results of the VS are reported in Table 3, after the deletion of those molecules which, although being part of the top 2% during the docking step, left the binding during the MD simulation (namely, GHRP-2, cobicistat, oxytocin, and vitamin B12). Although different Nwat values have been evaluated, we will limit our discussion to the results provided by Nwat-MMGBSA with Nwat = 60, since this value resulted to be appropriate when dealing with PPI inhibitors.^20,34^

**Table 3:** Results of the VS Campaign on the Crystal Structure of SARS-CoV-2 S-Protein RBD Binding Site 1^a^

Drug name	Dock score	Nwat-MMGBSA^b^
Polymyxin B	–107.6	–152.1 ± 11.5
Colistin	–101.7	–149.4 ± 12.5
Daptomycin	–95.2	–137.8 ± 13.1
Oritavancin	–93.6	–126.8 ± 13.3
Thymopentin	–92.4	–121.9 ± 14.6
Terlipressin	–103.7	–118.0 ± 9.6
Lypressin	–103.2	–111.3 ± 12.6
Vancomycin	–96.2	–104.6 ± 17.9
Leuprolide	–110.7	–101.3 ± 10.8
Alarelin	–104.6	–98.3 ± 8.9
Deferoxamine	–90.8	–97.4 ± 9.0
Bacitracin	–93.9	–97.0 ± 11.8
Sennoside B	–91.3	–94.9 ± 8.9
Angiotensin II	–104.2	–94.8 ± 11.6
Salvianolic acid B	–104.5	–93.9 ± 10.2
Gonadorelin	–104.2	–93.5 ± 8.7
Nafarelin	–111.8	–90.3 ± 11.5
Amphotericin B	–108.0	–89.2 ± 11.8
Madecassoside	–96.0	–88.5 ± 10.2
Micafungin	–95.6	–86.0 ± 12.4
Mupirocin	–91.4	–82.1 ± 7.0
Goserelin	–107.9	–81.0 ± 12.7
Nystatin	–102.2	–78.8 ± 12.8
Echinacoside	–93.2	–71.9 ± 9.5
Dalbavancin	–90.7	–69.7 ± 12.5
Tyloxapol	–106.6	–68.5 ± 7.7
Icatibant	–115.8	–67.9 ± 10.4
Landiolol	–91.6	–67.3 ± 12.4
Venetoclax	–92.5	–66.9 ± 7.3
Vilanterol	–95.1	–65.5 ± 8.7
Montelukast	–97.6	–65.3 ± 11.9
Salmeterol	–100.4	–64.6 ± 7.5
Ginsenoside Rb1	–96.2	–64.2 ± 10.9
Somatostatin	–98.6	–61.2 ± 10.2
Ledipasvir	–97.4	–60.8 ± 8.5
Zafirlukast	–91.0	–57.7 ± 6.0
Latanoprost	–98.0	–56.5 ± 7.4
Fexofenadine	–91.0	–53.3 ± 17.9
Velpatasvir	–91.3	–53.1 ± 8.7
Nebivolol	–90.9	–52.2 ± 7.5
Azelnidipine	–91.1	–51.6 ± 7.6
Astemizole	–91.9	–51.1 ± 5.5
Pranlukast	–91.5	–50.3 ± 5.6
Travoprost	–89.9	–49.1 ± 8.5
Vilazodone	–97.0	–48.6 ± 5.9
Aclidinium	–90.0	–48.5 ± 6.4
Octenidine	–102.4	–48.1 ± 9.5
Elbasvir	–97.4	–47.8 ± 10.0
L-Ascorbyl 6-palmitate	–90.8	–47.7 ± 8.0
Silodosin	–90.3	–47.1 ± 9.2
Ponatinib	–96.6	–44.6 ± 7.7
Ebastine	–95.2	–44.3 ± 7.9
Vitamin K2	–95.7	–41.5 ± 6.1
Posaconazole	–99.5	–32.6 ± 7.7
Penfluridol	–90.3	–31.5 ± 9.1
Vitamin A	–96.6	–31.1 ± 8.1
Lapatinib	–100.8	–31.1 ± 7.2
Behenic alcohol	–93.4	–28.8 ± 7.2
Gefarnate	–89.8	–26.2 ± 10.4
Azilsartan	–90.2	–24.5 ± 12.4

a Top 2% of compounds selected from the docking of 3118 FDA approved drugs and rescored by Nwat-MMGBSA are shown ranked by Nwat-MMGBSA scores.

b Energy obtained by using Nwat = 60, ± standard deviation.

It is not surprising that the best ranked ligands are peptide-like molecules, since these are usually larger than small molecules and allow a better interaction with the PPI binding partner. Most of the top ranked peptide-like molecules, such as the polymyxin B, colistin, and daptomycin, are currently used as antibiotics because of their ability in disrupting the bacterial membrane. Among these, polymyxin B has been tested within a compassionate use protocol for patients with an immediately life-threatening condition.^70^

Others compounds identified herein as potential binders of the S-protein are terlipressin and lypressin, analogs of vasopressin and used against hypotension. We can also find hormone-peptides, such as alarelin or leuprorelin, which belong to the gonadotropin-releasing hormone family, or somatostatin, an endocrine system regulator. Furthermore, we can observe the presence of peptidomimetics, such as icatibant, which acts as an antagonist of B2 bradykinine receptors. This last compound was also identified by an independent study as a potential disruptor of the S-protein-hACE2 PPI.^71^

The only non-peptide molecules found in the top 2% are large compounds (molecular weight >500 g/mol) rich in H-bond acceptor and donor atoms. This is not surprising, indeed it has been suggested that the SARS-CoV-2 binds to the host heparan sulfate chains of the heparan sulfate proteoglycan receptor also, initiating the internalization.^72^ In addition, it has been recently shown that the RBD can bind to the heparin^73−75^ and that an octosaccharide sequence strongly inhibits this interaction (IC_50_ = 38 nM).^73^ In our VS campaign, we found the salvianolic acid B (also found in M^pro^ screening), used as antioxidant, antifungal drugs (amphotericin B, micafungin, nystatin, micafungin), the madecassoside and ginsenoside Rb1, molecules with anti-inflammatory properties, and the tensioactive tyloxapol in the top 2%. Interestingly, a combination of amphotericin B and deoxycholate was shown to have an effect in decreasing the infectivity of transmissible gastroenteritis coronavirus.^76^ We also found at #11 deferoxamine, a chelating agent under clinical study against COVID-19 (ClinicalTrials.gov Identifier: NCT04333550). However, this molecule is claimed as responsible of chelating the iron whose dissociation from heme is increased by SARS-CoV-2, causing oxidative stress and damage to the lung.^77^ The top 2% also contains antivirals such as ledipasvir and elbasvir, used against hepatitis C. However, their mechanism of action involves the inhibition of viral proteins. Additionally, both elbasvir and ledipasvir were identified on the same target in another independent study, using a different computational protocol.^47^

Unexpectedly, among the best 60 RBD ligands there is salmeterol and vilanterol, which are agonists of β2-adrenergic receptors, a class of molecules which showed a minor amplification of the viral phenotype in a recent preprinted study.^78^ Conversely, another antiasthmatic drug, montelukast, has been shown to cause the disruption of the viral integrity of the Zika virus;^79^ thus, its presence in the top 2% (#39) enhances the interest of this compound against SARS-CoV-2 also.

In addition, we also found a few beta-adrenergic blockers, namely landiolol and nebivolol; this class of molecules, although it is not known to bind to the S-protein, has been hypothesized to be able to decrease the SARS-CoV-2 entry into the cells by downregulating ACE2 receptors.^78,80^

Peptides are usually highly flexible and require an extensive conformational sampling before the VS procedure. However, most of the peptides herein considered are partially cyclic: this creates a structural constraint, making feasible and globally reliable their docking to RBD.

**Figure 4:**
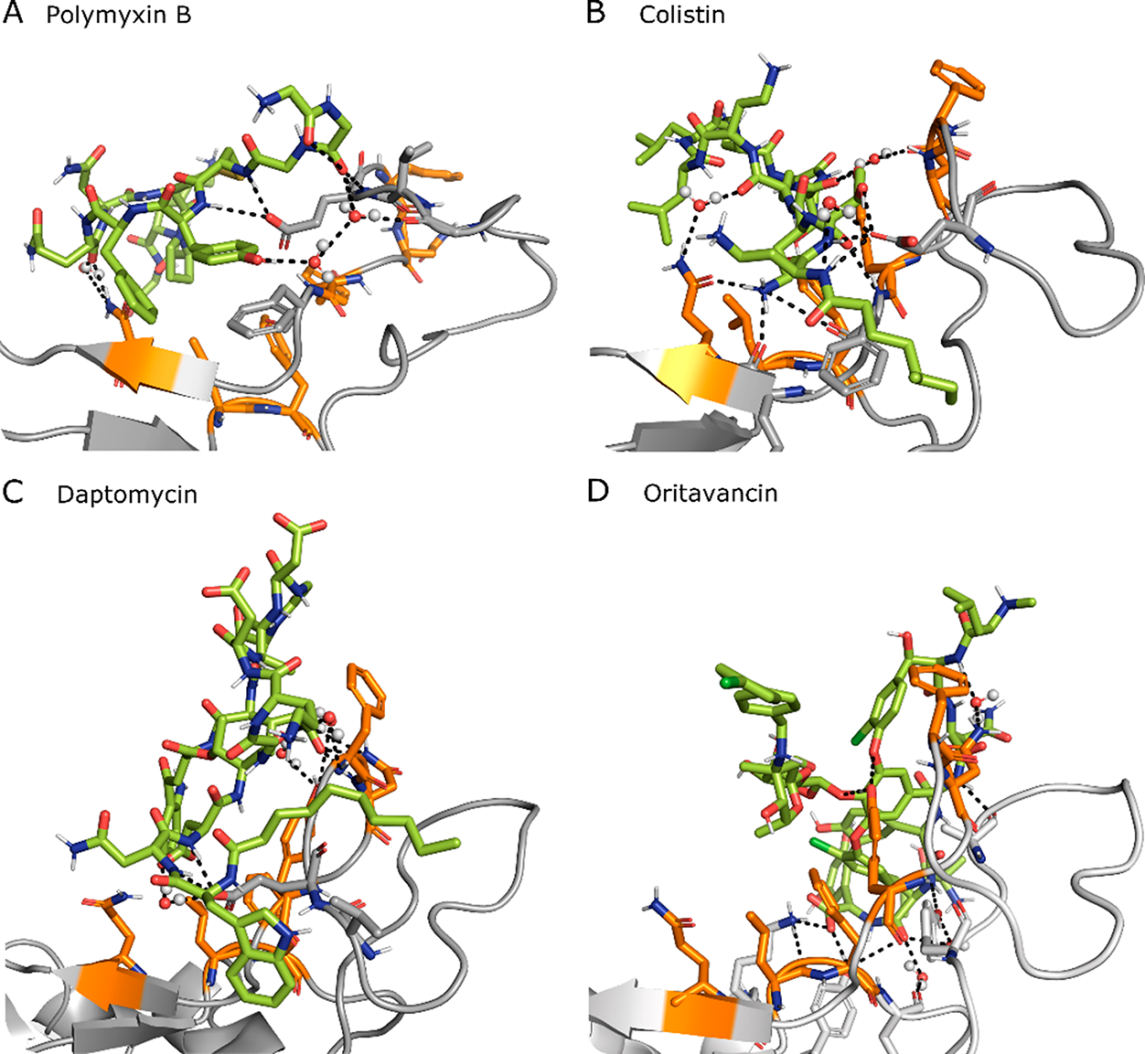
Snapshot of the MD simulation between RBD (gray) and one of the top four ligands (green). RBD BS1 hot spots are highlighted in orange. Direct and water (spheres) mediated H-bonds are also displayed as dashed lines. Additional RBD residues interacting with the ligand are displayed as sticks.

The best scored compounds interact with the RBD through hydrophobic interactions and stable direct and water-mediated H-bond with BS1 hot spots and neighboring residues, creating a stable network of interactions, as shown in Figure 4 for the four top-ranked ligands. For example, polymyxin B can create direct H-bonds with Glu484, Phe486, Asn487, Tyr489, and Gln493 and water-mediated H-bonds with Asn487, Glu484, and Phe490. In addition, we can observe hydrophobic interactions between polymyxin B and Val483 and Phe486.

### Virtual Screening on RBD-BS2

Similar results were obtained for the BS2, as shown in Table 4. In this case also, the ligands which left the binding site during the MD simulation, namely colistin, ritonavir, salmeterol, dalbavancin, and atazanavir, were removed from the list. It is interesting to notice that colistin was the second best ligand for BS1; conversely for BS2, even if the docking procedure ranked this ligand in the top 2%, it could not maintain the favorable interactions during the MD simulation. This highlights the importance of performing MD simulations on the complexes obtained by the docking procedure and provides a further confirmation of the quality of our protocol.^20^ In addition, although the amino acid composition of the two binding sites is quite similar (Figure 2), their conformational organization is specific and exploitable for further studies on the development of new potential inhibitors of the RBD-hACE2 interaction.

**Table 4:** Results of the VS Campaign on the Crystal Structure of SARS-CoV-2 S-Protein RBD Binding Site 2^a^

Drug name	Dock score	Nwat-MMGBSA^b^
Polymyxin B	–99.4	–164.3 ± 11.3
Thymopentin	–97.7	–154.5 ± 12.9
Icatibant	–107.6	–143.1 ± 12.0
Octreotide	–94.6	–127.2 ± 10.9
Oritavancin	–98.3	–123.6 ± 14.1
Nystatin	–110.8	–123.2 ± 10.5
Terlipressin	–98.2	–122.8 ± 10.7
Salvianolic acid B	–112.0	–121.6 ± 10.6
Echinacoside	–104.6	–113.3 ± 8.2
Bleomycin	–103.4	–110.1 ± 15.3
Angiotensin II	–100.3	–107.3 ± 12.1
Nafarelin	–121.9	–106.4 ± 10.6
Leuprorelin	–114.5	–106.2 ± 9.8
Sennoside B	–91.9	–99.1 ± 10.6
Aliskiren	–99.6	–96.3 ± 6.7
Caspofungin	–99.1	–95.4 ± 14.3
Alarelin	–103.7	–94.6 ± 10.3
GHRP-2	–104.9	–93.8 ± 9.9
Lentinan	–96.5	–93.4 ± 12.5
Leuprolide	–109.6	–93.4 ± 11.1
Hederacoside C	–98.5	–89.1 ± 10.1
Gonadorelin	–111.4	–88.8 ± 13.0
Pneumocandin	–95.3	–86.4 ± 11.4
Daptomycin	–94.4	–85.4 ± 18.5
NAD+	–96.9	–83.6 ± 33.4
Deferoxamine	–97.2	–83.3 ± 8.5
Goserelin	–99.2	–80.4 ± 10.9
Neohesperidin	–94.2	–79.8 ± 8.0
Gramicidin	–98.5	–79.3 ± 11.8
Somatostatin	–110.7	–77.2 ± 10.7
Vilanterol	–96.3	–75.5 ± 6.3
Desmopressin	–95.1	–74.9 ± 11.7
Elbasvir	–108.7	–73.4 ± 7.3
Manidipine	–92.6	–72.3 ± 6.4
Ginsenoside Rb1	–93.8	–72.3 ± 10.8
Lercanidipine	–95.5	–71.3 ± 6.5
Atazanavir	–98.1	–70.8 ± 7.4
Cobicistat	–100.3	–69.5 ± 8.7
Montelukast	–100.8	–67.5 ± 7.6
Vitamin B12	–93.5	–65.9 ± 11.7
Tyloxapol	–104.1	–64.5 ± 7.1
Micafungin	–95.4	–63.2 ± 12.7
Salmeterol	–99.8	–62.8 ± 8.6
Zafirlukast	–94.6	–61.8 ± 5.8
Labetalol	–91.8	–61.4 ± 5.9
Indinavir	–105.0	–60.0 ± 8.7
Latanoprost	–94.7	–57.2 ± 6.5
Amphotericin B	–132.7	–57.0 ± 7.8
Ombitasvir	–94.3	–53.2 ± 12.6
Tocofersolan	–91.6	–52.5 ± 6.8
Haloperidol	–91.9	–52.5 ± 9.2
Tafluprost	–94.3	–51.6 ± 6.3
Itraconazole	–96.0	–46.5 ± 7.3
Avanafil	–96.7	–46.2 ± 5.8
Ledipasvir	–92.6	–43.4 ± 8.2
Octenidine	–99.1	–43.2 ± 9.1
Thonzonium	–92.4	–41.0 ± 8.1
Fulvestrant	–96.5	–40.9 ± 7.1
Gefarnate	–91.7	–39.3 ± 6.6
Clindamycin	–91.9	–33.4 ± 7.8

a Top 2% of compounds selected from the docking of 3118 FDA approved drugs and rescored by Nwat-MMGBSA are shown ranked by Nwat-MMGBSA scores.

b Energy obtained by using Nwat = 60, ± standard deviation.

**Figure 5:**
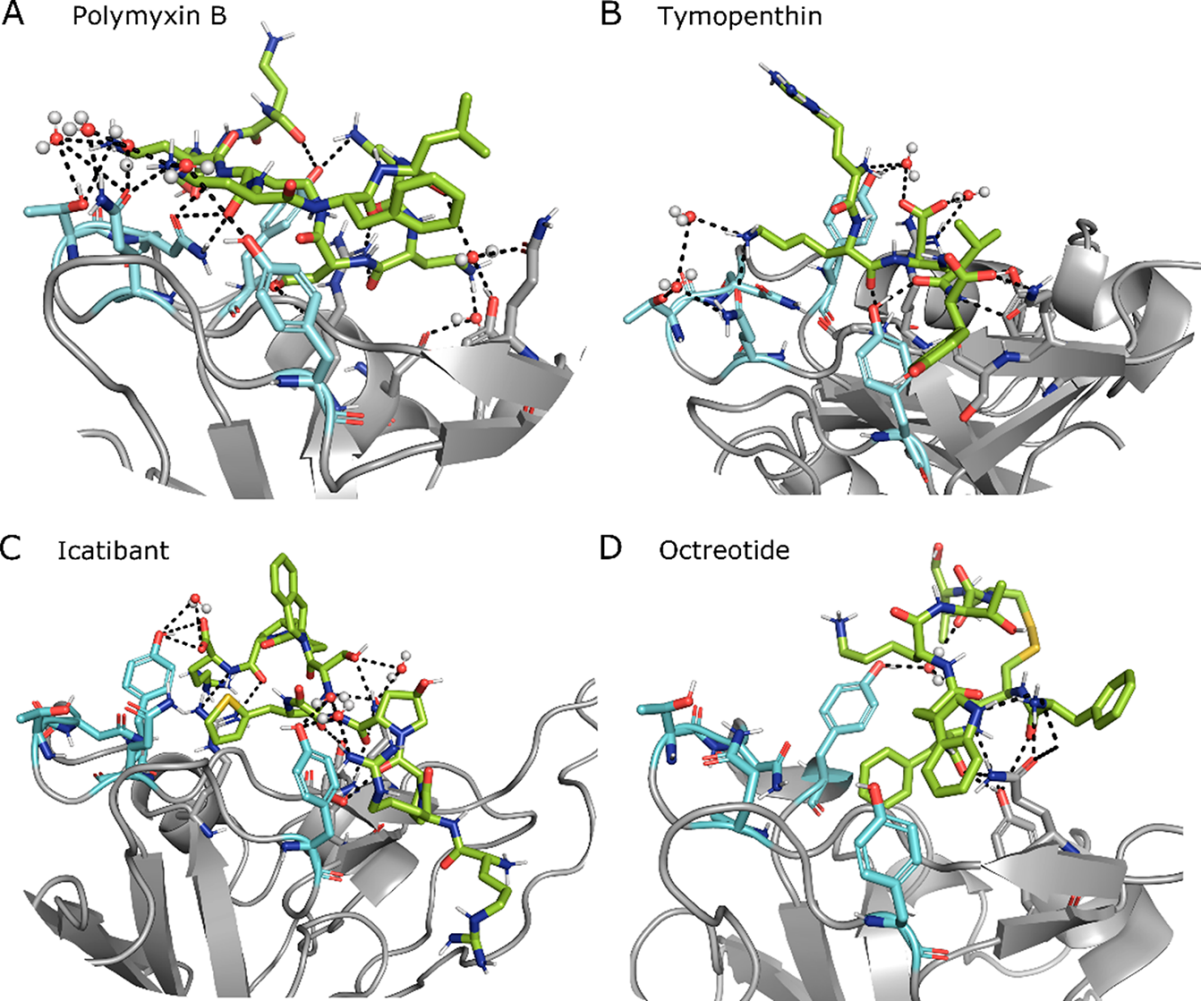
Snapshot of the MD simulation between RBD (gray) and one of the top four ligands (green). RBD BS2 hot spots are highlighted in cyan. Direct and water (spheres) mediated H-bonds are also displayed as dashed lines. Additional RBD residues interacting with the ligand are displayed as sticks.

Globally, the top 2% of ligands binding to BS2 is similar in composition to the one binding to BS1: most of the ligands are peptide-like molecules known to be antibiotics, antifungals, peptide hormones, and pressure regulators. As noticed for BS1, we can also find molecules containing both large hydrophobic groups and H-bond donors and acceptors, such as echinacoside and aliskiren (Table 4). The best ranked ligand poses show a tight network of direct and water-mediated H-bonds with both the hot spot residues and the neighboring ones, in addition to additional hydrophobic interactions (Figure 5). For example, the best ranked molecule, which is polymyxin B also in this case, creates direct H-bonds with Arg403, Tyr449, Gly496, the most relevant hot spot Gln498, and Asn501, together with water-mediated H-bonds with Glu406, Tyr449, Tyr453, Gln493, Gln498, and Thr500. Except for polymyxin B, the ranking is quite different from that for BS1, with molecules which were not present in the top 10 for BS1 being ranked in the top positions for BS2, such as icatibant (#3) and octeotride (#4).

Thymopentin is the only linear peptide found in the top 2% docked molecules for both BS. In order to verify if the docked conformation properly took into account for the peptide flexibility and its accessible conformations, we predicted the 3D structure of the peptide using the PEPFOLD3^81^ server, and the best model has a backbone RMSD of 1.3 Å (Figure S2) from the thymopentin docked to RBD and ranked second in the VS campaign targeting RBD BS2. It should be underscored that thymopentin is an immunostimulant peptide applied in numerous clinical studies during the AIDS pandemic between 1983 and 1985.^82,83^ Therefore, together with the good binding to the SARS-CoV-2 S-protein RBD shown within this VS campaign, a potential immunostimulant effect of this peptide could be helpful in enhancing the immune response to the viral infection.

## Conclusions

SARS-CoV-2 currently represents a major threat to human health, having caused hundreds of thousands of deaths in a few months. At the moment a cure against this pandemic infection is still lacking, together with a vaccine against this virus. In order to rapidly face the emergency, testing the efficacy against SARS-CoV-2 of drugs already approved for the treatment of other diseases (drug repurposing) is a good option. Indeed, it has the advantage of exploiting molecules which have already been tested in terms of toxicity and which are usually easy to purchase for clinical tests and patient administration. Therefore, positive results from this kind of procedure can speed up the process of finding a treatment against SARS-CoV-2. However, the number of approved drugs by the major jurisdictions is huge and directly performing either *in vitro* or *in vivo* studies on all of them would be time-consuming; thus a fundamental contribution to accelerate the screening can come from *in silico* techniques.

Within this context, we proposed a multiple VS campaign aimed to prioritize the testing against SARS-CoV-2 of already approved molecules. More in detail, we performed 4 independent VS procedures of more than 3000 approved drugs using two different SARS-CoV-2 proteins: the main proteinase M^pro^ and the RBD of the S-protein. Inhibiting the former would block the viral replication, while targeting the S-protein domain (i.e., RBD) would hamper the viral entry into the human cells. We applied an advanced VS procedure, which already proved to better discriminate between active and inactive compounds on multiple systems, compared to standard docking procedures.^20^

The VS campaign against M^pro^ ranked in the top 2% of inhibitors of the HIV protease, such as indinavir, atazanavir, and lopinavir, which recently proved to be able to alleviate the symptoms of mild-to-moderate SARS-CoV-2 infection in combination with ritonavir.^40^

The VS campaign on Spike protein RBD indicated that peptides or peptidomimetics actually used as antibiotics (i.e., polymyxin B, colistin, and daptomycin), pressure regulators (i.e., terlipressin and lypressin), hormone-peptides (i.e., alarelin and leuprorelin), and immunostimulants, such as the thymopentin, could be evaluated against SARS-Cov-2 also. Currently, there are not clinical studies on molecules known to specifically disrupt the interaction between the human ACE2 and the RBD; however, a few peptides were designed with this aim and successfully tested *in vitro*, validating our hypothesis that peptide-based molecules can be adapted to inhibit the ACE2-RBD interaction.

In conclusion, together with providing a good starting point for future *in vitro* and *in vivo* investigations on the resulting top compounds, the results of this extensive VS can support the design of selective and specific molecules to treat SARS-CoV-2 infection by targeting different viral proteins.

## Supporting Information Available

Figure S1. RMSD of the RBD-hACE2 complex backbone atoms from the X-ray structure during the MD simulation. Table S1. Difference in the binding free energies between the mutated and the native RBD-hACE2 complex obtained from the alanine scanning. Table S2. Compounds selected by docking on M^pro^ (6LU7 model) that failed during the MD/Nwat-MMGBSA rescoring step. Table S3. Compounds selected by docking on M^pro^ (QHD43415 homology model) that failed during the MD/Nwat-MMGBSA rescoring step. Table S4. Hydrogen bonds between RBD and hACE2 during the last half of the 20 ns MD simulation. Figure S2. Superposition of thymopentin docked to RBD BS2 and the PEPFOLD3 structure prediction. (PDF)

## Abbreviations

VS: virtual screening
MD: molecular dynamics
RBD: receptor binding domain
ACE2: angiotensin converting enzyme 2
H-bond: hydrogen bond
