# Supplementary material for: In Silico Drug Repurposing for SARS-CoV-2 Main Proteinase and Spike Proteins
Source: J Proteome Res. 2020 Sep 7;19(11):4637–48. doi: 10.1021/acs.jproteome.0c00383 (PMC7640956; doi:10.1021/acs.jproteome.0c00383)
Supplement: Supplementary file 1 — pr0c00383_si_001.pdf [file pr0c00383_si_001.pdf]

In silico drug repurposing for SARS-CoV-2 Main Proteinase and Spike proteins

Irene Maffucci[1,*], *Alessandro Contini*[2,*]

[1]Université de technologie de Compiègne, UPJV, CNRS, Enzyme and Cell Engineering, Centre de recherche Royallieu - CS 60 319 - 60 203 Compiègne Cedex

[2]Università degli Studi di Milano, Dipartimento di Scienze Farmaceutiche, Sezione di Chimica Generale e Organica "A. Marchesini", Via Venezian, 21 20133 Milano, Italy

Index

Figure S1. RMSD of the RBD-hACE2 complex backbone atoms from the X-ray structure (code PDB 6M0J) during the 20 ns MD simulation. S3

Table S1. Difference in the binding free energies between the mutated and the native RBD-hACE2 complex obtained from the alanine scanning performed on the last 10 ns of the MD simulation of the complex by individually mutating the RBD residues at the interface with hACE2. The numbering has been kept in according to the X-ray structure. S3

Table S2. Compounds selected by docking on M^{pro} (6LU7 model) that failed during the MD/Nwat-MMGBSA rescoring step. S4

Table S3. Compounds selected by docking on M^{pro} (QHD43415 homology model) that failed during the MD/Nwat-MMGBSA rescoring step........ S6

Table S4. Hydrogen bonds between RBD and hACE2 during the last half of the 20 ns MD simulation. Cut-offs of 3.5 Å and 150° have been used. The average donor-acceptor distances (AvgDist) are reported in Å and the average donor-donorH-acceptor angles are reported in degrees. S7

Figure S2. Superposition of thymopentin docked to RBD BS2 (light green) and the PEPFOLD3 structure prediction (dark green). The backbone RMSD between the two structures is 1.3 Å. ... S8

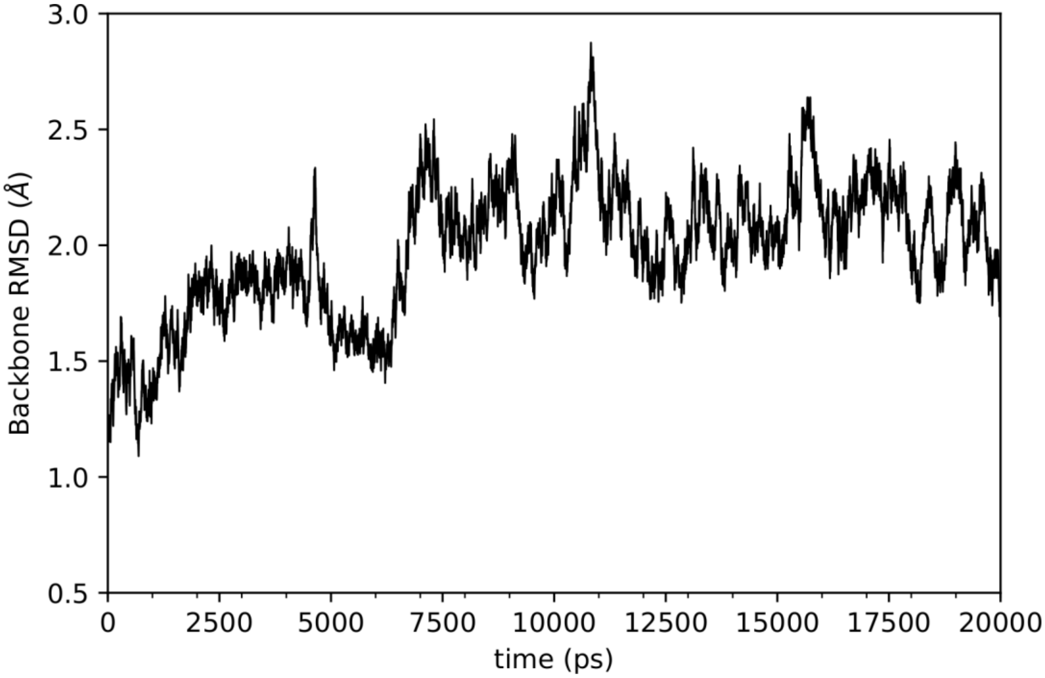

Figure S1. RMSD of the RBD-hACE2 complex backbone atoms from the X-ray structure (code PDB 6M0J) during the 20 ns MD simulation.

Table S1. Difference in the binding free energies between the mutated and the native RBD-hACE2 complex obtained from the alanine scanning performed on the last 10 ns of the MD simulation of the complex by individually mutating the RBD residues at the interface with hACE2. The numbering has been kept in according to the X-ray structure.

**#Residue**	**ΔΔG (kcal/mol)**	**Standard deviation (kcal/mol)**
R403	0.22	0.25
K417	2.23	2.17
Y449	2.74	1.35
Y453	0.10	0.60
L455	3.00	0.73
F456	3.57	1.06

Y473	0.40	0.24
S477	0.22	1.55
T478	-0.07	0.10
E484	-0.11	0.12
F486	4.45	1.55
N487	4.02	1.49
Y489	3.29	0.72
F490	-0.09	0.05
L492	0.07	0.03
Q493	4.75	1.89
S494	0.06	0.08
Y495	-0.08	0.05
Q498	9.13	3.43
T500	2.58	1.27
N501	4.25	1.44
V503	-0.01	0.09
Y505	4.64	1.81

Table S2. Compounds selected by docking on M^{pro} (6LU7 model) that failed during the MD/Nwat-MMGBSA rescoring step.

Drug Name	Dock score	MD/Nwat-MMGBSA
Nafarelin	-155.4	F
Leuprorelin	-145.6	F
Leuprorelin	-140.9	F
Somatostatin	-139.6	F
Icatibant	-139.3	F

Nystatin	-132.7	F
Goserelin	-128.8	F
Alarelin	-128.5	F
Gonadorelin	-125.5	F
Amphotericin B	-124.7	F
Carfilzomib	-122.4	F
Thymopentin	-121.2	F
Lentinan	-116.4	F
Ritonavir	-116.1	F
NAD+	-114.1	F
Octreotide	-113.6	F
Colistin	-113.5	F
Cangrelor	-111.6	F
Oxytocin	-111.4	F
Flucytosine	-111.3	F
Echinacoside	-110.3	F
Monomethyl auristatin E	-110.2	F
Salmeterol	-110.1	F
Daptomycin	-109.9	F
Pneumocandin B0	-109.6	F
Caspofungin	-109.1	F
Vilanterol	-109.1	F
Stachyose	-108.2	F
Ginsenoside Rb1	-108.1	F
Venetoclax	-107.7	F
Terlipressin	-106.7	F
Lopinavir	-106.7	F

Atracurium besylate	-106.5	F
Polymyxin B	-106.5	F
Deferoxamine mesylate	-106.2	F
Darunavir	-105.9	F

Table S3. Compounds selected by docking on M^{pro} (QHD43415 homology model) that failed during the MD/Nwat-MMGBSA rescoring step.

Drug Name	Dock score	MD/Nwat-MMGBSA
Gonadorelin	-131.4	F
Leuprorelin	-127.9	F
Nafarelin	-127.9	F
Leuprorelin	-124.1	F
Goserelin	-117.8	F
Bacitracin	-117.0	F
Amphotericin B	-115.2	F
Alarelin	-114.6	F
Deferoxamine	-113.7	F
Nystatin	-112.8	F
Octreotide	-108.0	F
Carfilzomib	-107.7	F
Terlipressin	-107.3	F
Somatostatin	-106.5	F
Flucytosine	-106.4	F
Lypressin	-106.3	F
Sennoside A	-105.6	F
Colistin	-103.9	F

Polymyxin B	-103.3	F
Nelfinavir	-102.7	F
Madecassoside	-102.5	F
Asiaticoside	-102.4	F
Pneumocandin B0	-102.4	F
Stevioside	-101.4	F
Thymopentin	-100.4	F
Tenofovir Disoproxil Fumarate	-100.3	F
Lapatinib	-99.3	F
Octreotide	-99.2	F
Desmopressin	-99.1	F
NAD+	-98.5	F

Table S4. Hydrogen bonds between RBD and hACE2 during the last half of the 20 ns MD simulation. Cut-offs of 3.5 Å and 150° have been used. The average donor-acceptor distances (AvgDist) are reported in Å and the average donor-donorH-acceptor angles are reported in degrees.

#Acceptor	DonorH	Donor	Frac	AvgDist	AvgAng
LYS_353@O	GLY_502@H	GLY_502@N	0.9026	2.8952	164.4105
ASN_487@OD1	TYR_83@HH	TYR_83@OH	0.6796	2.7777	162.7078
ASP_355@OD2	THR_500@HG1	THR_500@OG1	0.5978	2.7536	163.9769
GLU_35@OE2	GLN_493@HE22	GLN_493@NE2	0.5772	2.88	164.7713
ASP_38@OD1	TYR_449@HH	TYR_449@OH	0.4672	2.721	164.7831
ASP_38@OD2	TYR_449@HH	TYR_449@OH	0.4454	2.7187	164.3915
ASP_38@OD2	GLN_498@HE22	GLN_498@NE2	0.4424	2.9078	164.7138
ASP_38@OD1	GLN_498@HE22	GLN_498@NE2	0.3782	2.8983	164.6202

GLU_37@OE2	TYR_505@HH	TYR_505@OH	0.3434	2.7076	163.8788
GLU_35@OE1	GLN_493@HE22	GLN_493@NE2	0.2426	2.8978	164.91
TYR_41@OH	THR_500@HG1	THR_500@OG1	0.2272	2.8517	163.2088
ASP_30@OD2	LYS_417@HZ3	LYS_417@NZ	0.218	2.774	162.3672
ASP_30@OD2	LYS_417@HZ1	LYS_417@NZ	0.2116	2.7782	161.6638
GLN_498@OE1	LYS_353@HZ3	LYS_353@NZ	0.1462	2.8426	161.4818
GLN_498@OE1	LYS_353@HZ2	LYS_353@NZ	0.1402	2.8527	161.641
ASP_30@OD2	LYS_417@HZ2	LYS_417@NZ	0.1324	2.7924	162.3512
GLN_498@OE1	LYS_353@HZ1	LYS_353@NZ	0.1298	2.8405	161.1518
GLN_493@OE1	LSY_31@HZ1	LSY_31@NZ	0.1262	2.8247	162.1882
GLN_493@OE1	LSY_31@HZ3	LSY_31@NZ	0.1252	2.8188	161.9295
GLU_37@OE1	TYR_505@HH	TYR_505@OH	0.1176	2.7326	163.3618
GLN_493@OE1	LSY_31@HZ2	LSY_31@NZ	0.1168	2.8314	162.1823
GLN_24@OE1	ASN_487@HD21	ASN_487@ND2	0.1118	2.9744	159.9545
GLN_24@OE1	SER_477@H	SER_477@N	0.1114	2.9675	163.4158

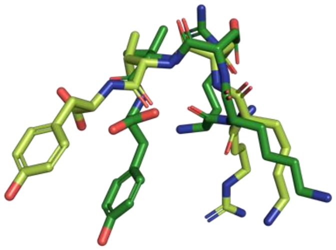

Figure S2. Superposition of thymopentin docked to RBD BS2 (light green) and the PEPFOLD3 structure prediction (dark green). The backbone RMSD between the two structures is 1.3 Å.
